# Updates in Refractory Status Epilepticus

**DOI:** 10.1155/2018/9768949

**Published:** 2018-05-08

**Authors:** Rohit Marawar, Maysaa Basha, Advait Mahulikar, Aaron Desai, Kushak Suchdev, Aashit Shah

**Affiliations:** Department of Neurology, Detroit Medical Center and Wayne State University, Detroit, MI 48201, USA

## Abstract

Refractory status epilepticus is defined as persistent seizures despite appropriate use of two intravenous medications, one of which is a benzodiazepine. It can be seen in up to 40% of cases of status epilepticus with an acute symptomatic etiology as the most likely cause. New-onset refractory status epilepticus (NORSE) is a recently coined term for refractory status epilepticus where no apparent cause is found after initial testing. A large proportion of NORSE cases are eventually found to have an autoimmune etiology needing immunomodulatory treatment. Management of refractory status epilepticus involves treatment of an underlying etiology in addition to intravenous anesthetics and antiepileptic drugs. Alternative treatment options including diet therapies, electroconvulsive therapy, and surgical resection in case of a focal lesion should be considered. Short-term and long-term outcomes tend to be poor with significant morbidity and mortality with only one-third of patients reaching baseline neurological status.

## 1. Introduction

Status epilepticus (SE) is a neurologic emergency associated with significant morbidity and mortality. It is seen across all ages, and around 200,000 cases are seen in the United States annually [1].

Status epilepticus is defined as persistent or recurrent seizures due to a failure of seizure termination mechanisms. In bilateral tonic-clonic seizures, it has been well accepted that 5 minutes of seizure activity constitutes status and has been shown that long-term consequences begin at 30 minutes of status. Similar data are lacking for focal status epilepticus. However, recently, the International League Against Epilepsy (ILAE) has proposed 10 minutes' duration as a time point for which focal status epilepticus can be defined (termed “point t1”) and 60 minutes for which long-term consequences may occur in focal status (termed “point t2”) [2]. These are arbitrary terms which lack substantial evidence in the case of focal SE.

Refractory status epilepticus (RSE) occurs when seizures persist despite administration of one first-line medication (IV benzodiazepine) and one second-line medication (IV antiepileptic drug) [3]. Super-refractory status epilepticus (SRSE) is defined as SE that persists despite 24-hour treatment with IV anesthetic and recurs when weaning the patient off the anesthetic [4]. New-onset refractory status epilepticus (NORSE) is defined as new-onset RSE where no discernible cause is identifiable in otherwise healthy individuals [5, 6].

SE is classified based on semiology and clinical manifestations. Trinka et al. proposed semiology as axis 1 of classification of SE. These are broadly differentiated into those with and without prominent motor symptoms. Those with prominent motor symptoms are further divided into convulsive (generalized and focal to generalized), myoclonic, or focal. SE without prominent motor symptoms is termed “nonconvulsive status epilepticus” either with or without coma. The distribution of convulsive and nonconvulsive SE varies widely across different studies [2].

The scope of this review is to primarily provide updates in management of refractory status epilepticus. With this aim, we focused on adult RSE cases. We also tried to exclude the common etiology of anoxic brain injury as it has significantly different managements and outcomes. Given that some status epilepticus research does not clearly differentiate between SE, RSE, and SRSE, some of the matter here will also apply for SE and SRSE.

## 2. Epidemiology

The incidence of status epilepticus ranges from approximately 5 to 40 per 100,000 based on several population-based studies across the US, Europe, and Asia with a recent meta-analysis reporting an annual incidence of 12.6 per 100,000 [7–9]. There is no significant difference in the incidence of SE in males and females. However, the annual incidence in elderly of 27.1 per 100,000 is approximately four times that of nongeriatric adults. There is no difference in the incidence in developing and developed countries. The more severe and prolonged types of SE are refractory status epilepticus (RSE) and super-refractory status epilepticus (SRSE). RSE occurs in 29 to 43% of SE cases, and SRSE in seen in 12 to 26% of SE cases and 13 to 42% of RSE cases.

## 3. Etiology

Of SE episodes, 29 to 43% will develop into RSE in retrospective studies [3, 12, 15–20]. One large prospective study and one small prospective study show incidence at the lower spectrum of the above range at 33% and 31%, respectively [10, 21]. The etiology of RSE can broadly be categorized into those with existing epilepsy and those with no known history of epilepsy. New-onset refractory status epilepticus (NORSE) could be of unknown cause (idiopathic or cryptogenic being other commonly used terms) or secondary to an inflammatory etiology [5].

An accepted etiological classification of RSE is not available. However, ILAE does broadly break down the etiology of SE into known and unknown as mentioned in Table 1. Known can be further differentiated into acute, remote, and progressive and as part of electroclinical syndromes. Some studies have used this classification as shown in Table 2. An acute symptomatic etiology is the predominant cause accounting for 41 to 77% of the cases. In two studies, the acute symptomatic etiology reached statistical significance as the most common cause of RSE as compared to nonrefractory status epilepticus (NRSE) [3, 10]. One study showed that the remote symptomatic etiology was more likely with NRSE as compared to RSE [21].

A more exhaustive list of SE etiologies is also provided by ILAE (Table 1) [2]. These etiologies are also applicable for RSE. Some other studies have described etiologies in this format (Table 3). When specific etiologies are considered, CNS infections, especially encephalitis, are a frequent cause. Neurocysticercosis is the leading cause of epilepsy in developing countries and worldwide. However, it is likely an uncommon etiology of SE occurring in less than 10% of cases [22]. Interestingly, in one study, anoxic brain injury was the reason in 50% of cases, but no CNS infections were found. Two studies showed encephalitis as a statistically significant most common etiology at 22% and 31%, respectively [3, 12]. Other commonly noted etiologies include unknown, immunological, and cerebrovascular (including hemorrhages). Most studies do not break down cases into those with new onset versus seizure versus established epilepsy. Regardless, missing AEDs is not an insignificant reason for RSE accounting for up to 16% of cases. One study found substance abuse as more likely to be associated with NRSE than RSE [3]. Specific studies mentioned in Tables 2 and 3 excluded anoxic brain injury as an etiology [11, 23]. Etiology is usually singular, but a significant minority can have multiple etiologies. As per an international audit, 13% of patients had two or more etiologies [24]. One study showed nonconvulsive status epilepticus (NCSE) or focal motor seizures at onset as independent risk factors for RSE [19]. Specifically, NORSE has a different distribution of etiologies with the most common being unknown, while a significant number (37%) tend to be secondary to paraneoplastic or autoimmune pathologies [5].

## 4. Investigations

### 4.1. Overview

The management of SE is challenging, and establishing an etiology is integral to the treatment of SE. In most cases, the etiology is known, with the usual culprits being previous seizures, intracranial lesions, and infections. However, in cases of refractory and super-refractory status epilepticus, it is often difficult to ascertain a cause.

The initial investigation should be done within minutes of patient arrival and should be inclusive of but not limited to venous blood for analyzing electrolytes, liver function tests, glucose, complete blood count, AED levels (in case of known history of epilepsy), and other drug levels or toxicological screens (e.g., in young patients with new-onset seizures). This should be followed up with computerized tomography of the head as soon as the patient is stable to look for any structural lesion(s) or any acute intracranial lesions like hemorrhages and hematomas that might need emergent intervention. In patients with fever and sudden-onset altered mental status, there should be a low threshold to perform a lumbar puncture to rule out common infections especially herpes encephalitis. An emergent EEG should be considered in cases of prolonged seizures and if the patient is not back to baseline soon to look for NCSE. Consider testing for metabolic and mitochondrial diseases in a young adult with known myoclonus, intellectual disability, and other unexplained neurological and systemic symptoms and signs. Proposed workup in identifying the etiology of RSE if a discernible cause is not apparent with initial testing is described in detail in Table 4 [25]. More detailed discussion on EEG, autoimmune testing, and neuroimaging is provided below.

### 4.2. EEG

Electroencephalography (EEG) is used to detect and later manage SE. EEG criteria for the diagnosis of SE include frequent repetitive electrographic seizures and repetitive generalized or focal epileptiform discharges of greater than 3 Hz. Repetitive or periodic epileptiform discharges less than 3 Hz can be considered ictal if associated with an improved clinical response with repeated short treatment with a benzodiazepine. Without a clear response, such EEG patterns fall along the ictal-interictal continuum without clear indication or consensus for continued treatment [26].

Patients who are treated after convulsive SE and who go on to have persistent coma for two hours or more develop NCSE in 13 to 48% of cases reviewed [27, 28]. Patients with an underlying brain pathology are more likely to develop NCSE after convulsive SE, while patients with AEDs or alcohol withdrawal are less likely to develop NCSE. Patients who are critically ill with a depressed level of consciousness were found to have NCSE in 8% of cases despite no prior seizures [29]. In about half of the cases, seizures are captured within the first hour of EEG recording [30], and in the comatose, it can take 24 to 48 hours to capture seizures [31].

Continuous EEG (cEEG) is also required to help achieve treatment goals of seizure freedom versus the burst suppression pattern after IVAD administration is initiated [32]. In some instances, the reactivity of EEG to drug administration such as the development of frontal alpha after administration of ketamine has been proposed to be a possible indicator of success [33]. Automated and quantitative EEG (qEEG) software can be employed to aid in the detection of seizures and assessing burst suppression ratios using the color density spectral array and amplitude-integrated EEG. Although qEEG improves the reader time for the EEGer, sensitivity for seizure detection is decreased especially in short seizures with low amplitudes and slow frequencies [34, 35]. False-positive rates can also be high and average about one per hour when qEEG is used alone [34].

### 4.3. Autoimmune Investigations

Recently, an autoimmune etiology of status epilepticus is increasingly recognized. However, it remains an uncommon cause. Contrarily, it is becoming clear that, in certain circumstances, the autoimmune etiology should be suspected early. Early identification of immune-mediated disorder may lead to immune modulatory intervention early in the disease and improve the outcome. One of the vital presentations of autoimmune encephalitis is new-onset refractory status epilepticus or NORSE, which represents up to 40% of refractory convulsive status epilepticus [36]. Other syndromes, perhaps representing a similar spectrum of disorders, described primarily in children include febrile infection-related epilepsy syndrome (FIRES) or devastating epileptic encephalopathy in school-aged children (DESC). The following scenarios should heighten the suspicion of autoimmune etiology in patients with status epilepticus: (1) status epilepticus as presentation of new-onset seizures; (2) progression to refractory or super-refractory status epilepticus; (3) relatively recent but explosive onset of seizures; (4) the absence of established epilepsy history; (5) the presence of other neurological problems such as memory loss, autonomic or hypothalamic dysfunction, and ataxia or movement disorder; (6) new psychiatric symptoms or behavioral changes; (7) known history of cancer; and (8) lymphocytic pleocytosis on CSF examination [37].

Commonly associated autoantibodies to refractory status epilepticus are mentioned in Table 5 [38, 39]. Hashimoto encephalopathy and Rasmussen encephalitis are more distinct syndromes and often present with refractory status epilepticus. Hashimoto encephalopathy is associated with very high titers of anti-thyroid peroxidase (a-TPO) antibody and autoimmune thyroiditis, while Rasmussen encephalitis is thought to be a T-cell-mediated disorder, although various antibodies are found in this disorder [40]. Hashimoto encephalopathy responds well to corticosteroids in the majority and is also identified as steroid-responsive encephalopathy associated with autoimmune thyroiditis (SREAT) [41]. On the other hand, Rasmussen encephalitis does not respond well to medical management (except some response to IVIg) and often requires surgical intervention in the form of hemispherectomy to halt the progression of the disease and control status epilepticus.

Examination of CSF is helpful but shows nonspecific inflammatory changes with mild pleocytosis and elevation of protein. However, it can be normal in up to 40–50% of the patients. Other autoimmune inflammatory markers such as the presence of oligoclonal bands are typically absent. CSF abnormalities can also be transitory and may present in some samples but may not be present during same illness sampled at another time [5, 42]. Antibody testing in serum versus CSF is a difficult one to answer as no systematic review is available, and most studies are retrospective. In general, the presence of a specific antibody in CSF is given more weight in making a definite diagnosis. Likelihood of finding antibodies in CSF is higher compared to checking the serum titer in isolation in cases of anti-NMDA-R and anti-GABA_B_-R antibody syndromes [43, 44]. The higher antibody titer in the CSF compared to that of serum, especially higher than the IgG index, is considered a sign of intrathecal antibody synthesis and more likely to be associated with the autoimmune encephalitis.

### 4.4. Neuroimaging

**Figure 1:**
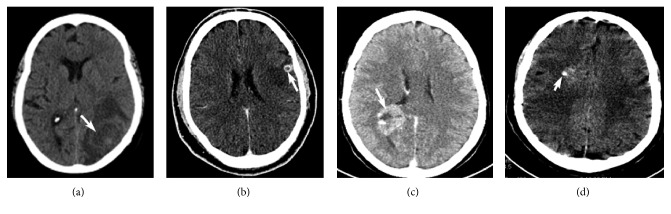
Emergent CT scan of the head obtained in the setting of new-onset recurrent seizures or status epilepticus showing various abnormalities. (a) A CT without contrast showing an area of a rounded lesion (arrow) with perilesional edema proven to be a cerebral abscess. (b) A postcontrast CT scan showing a small round enhancing lesion (arrow) with perilesional edema later proven to be neurocysticercosis. (c) A postcontrast CT showing a large enhancing heterogeneous mass (arrow) pathologically proven to be glioblastoma cerebrii. (d) A CT scan without contrast showing an area of calcifications (arrow) in arteriovenous malformation in a young man presenting with recurrent seizures.

**Figure 2:**
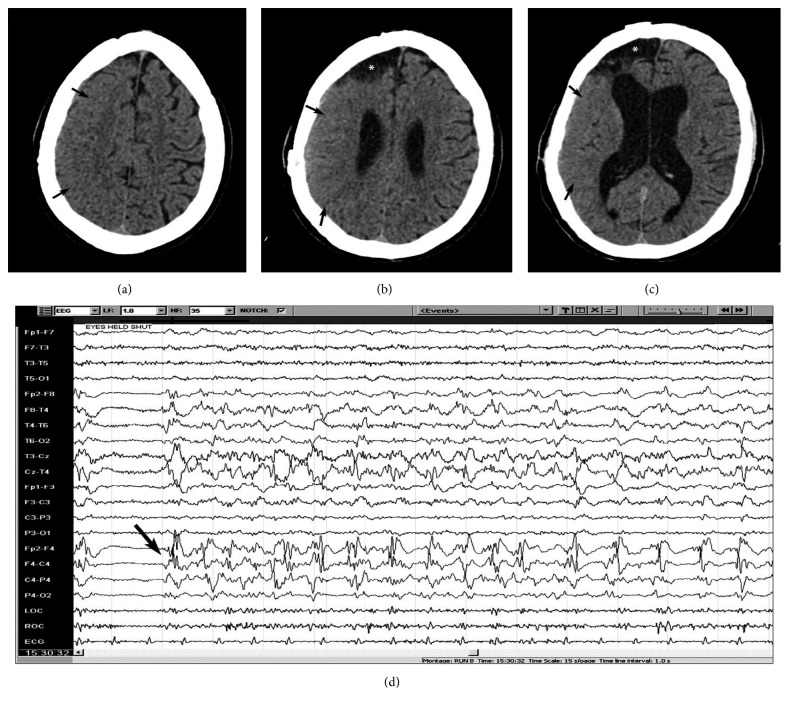
CT scan of the brain without contrast showing acute changes associated with status epilepticus. A middle-aged man with a history of alcoholism and previous traumatic brain injury with surgical intervention resulting in right frontal encephalomalacia presented with recurrent focal seizures consisting of head and eye deviation to the left and left upper extremity clonic activity. He developed new focal weakness of the left upper extremity and left hemianopia that recovered quickly with control of seizures, only to recur few days later with new confusion. An urgent CT scan of the head without contrast showed a large area with effacement of sulci and loss of gray-white differentiation involving the right frontal and parietal lobes (thin black arrows in (a), (b), and (c)), and EEG showed focal right frontal status epilepticus (thick black arrow in (d)). Also note an area of encephalomalacia involving the right anterior frontal lobe (asterisk in (b) and (c)).

Structural lesions can be responsible for seizures and status epilepticus; hence, structural neuroimaging can reveal abnormalities frequently. A CT scan can reveal either acute abnormalities or an old lesion in case of chronic epilepsy. The lesions that can be easily identified on cranial CT scan include intracranial hemorrhage, vascular malformation, brain tumor, stroke, abscess, or other infectious processes or even brain malformation (Figure 1). Brain MRI with a better definition of the brain structure is more sensitive in identifying structural lesions that might be responsible for epilepsy in the acute or chronic setting [45]. At times, the CT scan may show focal decreased attenuation with effacement of sulci and loss of gray-white differentiation in the area where the seizures originate (Figure 2).

**Figure 3:**
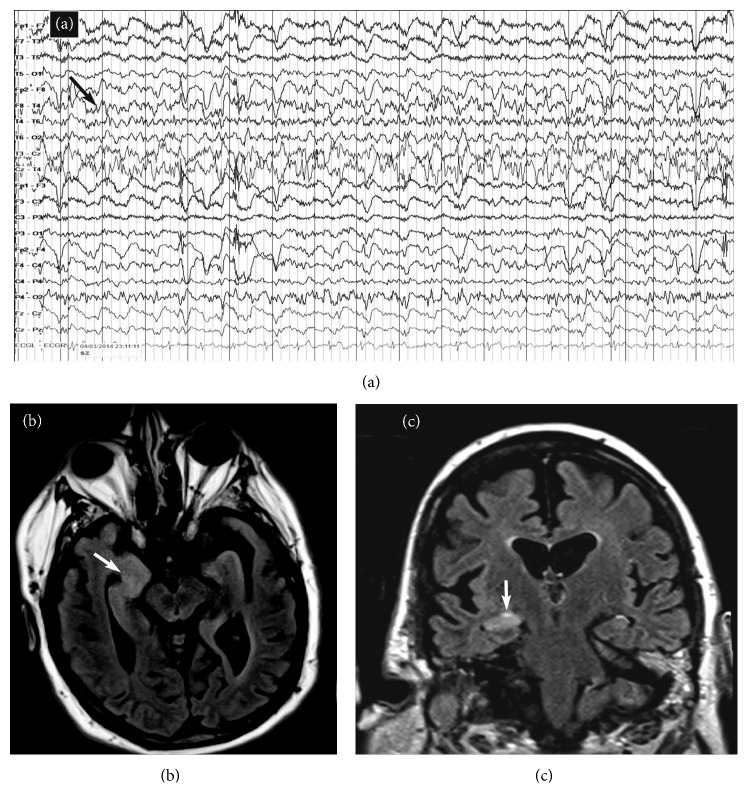
MRI changes associated with acute status epilepticus. A middle-aged man presenting with a previous history of epilepsy following a generalized tonic-clonic seizure. He failed to recover to baseline, and an urgent EEG was obtained that showed focal status epilepticus from the right temporal region (black arrow in (a)). MRI images obtained during the same admission showed an increased signal and swelling of the right hippocampus on axial (white arrow in (b)) and coronal (white arrow in (c)) FLAIR images.

**Figure 4:**
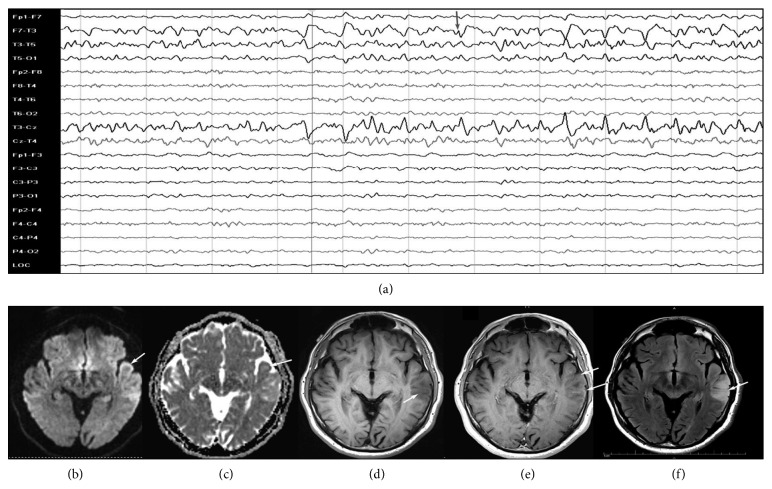
Various modalities of MR imaging showing changes associated with focal status epilepticus. A previously healthy middle-aged man presented with his first generalized tonic-clonic seizure followed by intermittent receptive dysphasia. Continuous EEG monitoring showed nonconvulsive status epilepticus originating from the left temporal leads (gray arrow in (a)). His MRI showed a focal area of abnormality involving the posterior superior aspect of the left temporal lobe. The DWI images showed a gyriform pattern of the increased signal (arrow in (b)), part of which showed decreased attenuation on an ADC map (arrow in (c)). The same area showed hypoattenuation on the T1W images with minimal pial surface enhancement (d, e) and increased signal with sulcal effacement on FLAIR images (arrow in (f)). The pathology showed neuronal necrosis, prominent reactive astrocytosis, microglial activation, and sparse mononuclear inflammation.

**Figure 5:**
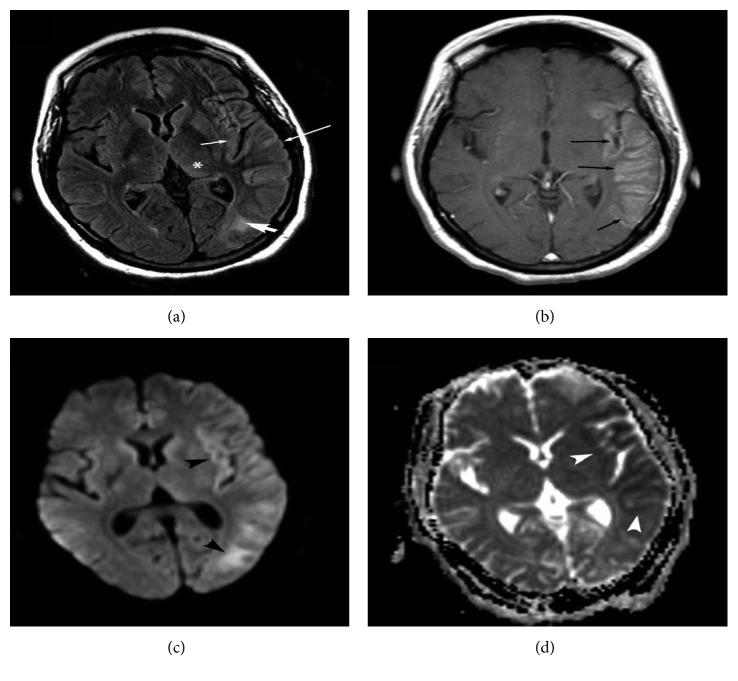
Selected MRI images from a woman with a new-onset focal refractory status epilepticus of the left temporal region. (a) A FLAIR image shows an increased signal involving the cortical gray matter with swelling of gyri of the temporal lobe, occipital lobe, and insula (thin white arrows in (a)). There are also areas of subcortical white matter hyperintensity (thick white arrow in (a)) and distal abnormality involving the posterior thalamus (pulvinar) (asterisk in (a)). (b) An axial postcontrast T1W image shows gyriform enhancement of the same region as FLAIR abnormalities (black arrows in (b)). (c) A diffusion-weighted image (DWI) shows an increased signal (black arrowheads in (c)). (d) An ADC map image shows decreased attenuation in the same region (white arrowheads in (d)) as DWI abnormalities suggestive of cytotoxic edema.

**Figure 6:**
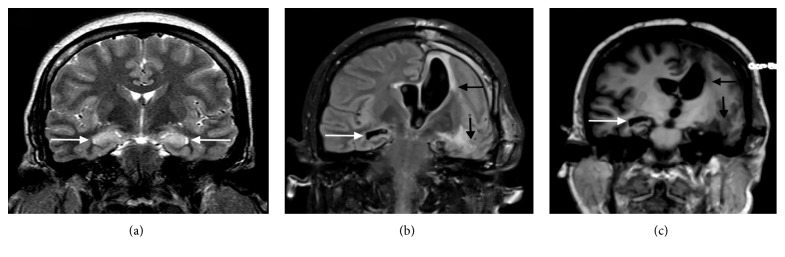
Long-term effect of status epilepticus. A previously healthy young woman presented with a new-onset refractory status epilepticus originating from the left hemisphere. Her initial MRI scan showed bilateral hippocampal swelling with an increased signal on the coronal FLAIR image (white arrows in (a)). Due to prolonged refractory status epilepticus, she underwent acute palliative resective surgery with removal of her dominant epileptic foci in the left frontal and temporal lobes. A repeat MRI four months later ((b) coronal FLAIR image and (c) noncontrasted T1W) showed postsurgical changes on the left (black arrows in (b) and (c)) with marked atrophy of the right hippocampus (white arrows in (b) and (c)).

On the other hand, specific, transient peri-ictal MRI abnormalities are reported following status epilepticus or cluster of seizures and are thought to be the direct result of recurrent seizures in a short time span. These changes are potentially caused by increased perfusion and metabolic activity due to ictal activity, postictal hypoperfusion, and transient ultrastructural pathologic alteration [46]. Commonly described findings include increased T2 fluid-attenuated inversion recovery (FLAIR) and diffusion-weighted imaging (DWI) signals, a variable degree of reduction in the apparent diffusion coefficient (ADC), and enlargement of the hippocampus ipsilateral to the seizure onset (Figures 3 and 4). Other patterns described include gyral distribution, T2 prolongation, and restricted diffusion involving the area of seizure origin or propagation (Figures 4 and 5). Less commonly seen abnormalities include patchy focal enhancement due to blood-brain barrier breakdown and increased vessel caliber/flow indicative of increased perfusion around the seizure origin (Figures 4 and 5). More distant abnormalities are also described, such as restricted diffusion affecting the splenium [47], unilateral or bilateral increased signal on T2 FLAIR imaging affecting the ipsilateral posterior thalamus/pulvinar region, or the contralateral cerebellum representing cerebellar diaschisis [48, 49] (Figure 5). The involvement of the pulvinar tends to occur less frequently compared to the cortical involvement and is associated with longer duration of SE suggesting the spreading pattern of seizure discharges. The location of the DWI and T2W changes correlates with the ictal onset but cannot be utilized as definitive seizure onset area as it can be seen in the distant areas of seizure spread in the epileptic network, for example, ipsilateral pulvinar. In animal models of status epilepticus, the severity of decrease in ADC maps correlated with the extent of neuronal loss [50]. The areas of increased T2 FLAIR and DWI signals in the acute phase may progress to show atrophy of the affected structure on subsequent MRI, suggesting that the initial abnormalities were indicative of the neuronal loss (Figure 6) [51–54]. It is likely that MRI changes are more common in patients with focal seizures, and EEG patterns often include lateralized periodic epileptiform discharges or intermittent seizure patterns with rhythmic epileptiform discharges and may also have a preexisting cortical lesion [52, 55].

Neuroimaging findings in autoimmune status epilepticus are variable and can be normal. If abnormal, they tend to show an increased signal on T2W or FLAIR images involving medial temporal lobe structures unilaterally or bilaterally. It may also show multifocal lesions involving the temporal neocortex, medial frontal/parietal and orbitofrontal lobes, or hypothalamus. Occasionally, contrast enhancement is seen in the same area suggesting disruption of the blood-brain barrier. These changes usually lag clinical onset and are present few days during the illness and represent cytotoxic edema with an increased signal on DWI images. Over time, repeat MRIs have shown atrophy of some of these structures [54, 56, 57].

## 5. Treatment

### 5.1. Existing Paradigm

The primary aim of treating a patient with SE is the rapid termination of the SE and aggressive management of an underlying acute symptomatic etiology. Left untreated, it can progress to RSE and SRSE. In a general sense, the longer the duration of untreated SE, the harder it is to treat [58, 59]. The Veterans Affairs Cooperative Study, one of the most significant studies of SE, showed that SE treatment becomes less effective with increasing duration of SE [10]. Notably, nonconvulsive status epilepticus is harder to treat and is controlled by AEDs in only 15% of cases compared to convulsive status epilepticus, in which up to 55% of cases may respond to the first AED [10]. Moreover, the short-term mortality rate of RSE is between 16 and 39% which is about three times higher than that of NRSE [3, 18, 19, 60].

The current guidelines for managing SE are not age-specific because the disease pathophysiology and the drug effects on neuronal receptors are the same in infants, children, and adults (though neonates may be the exception). They follow the sequential intravenous administration of three groups of drugs: (1) benzodiazepines aimed at rapid SE control; (2) classical AEDs targeted at early resistant forms and longer-term coverage; and (3) general anesthetics for SRSE.

Benzodiazepines (BZDs) act as positive allosteric modulators on gamma amino butyric acid (GABA) type A receptors [61]. A BZD in any form, either intravenous (IV), intramuscular (IM), or per rectal (PR), is recommended as the initial therapy of choice [62]. The commonly used BZDs are IM/IV midazolam and IV lorazepam or diazepam (PR in children). BZDs are more likely to work if used early, closer to seizure onset and decrease in effectiveness as seizure duration increases. This is because GABA receptors are internalized with time, and there is a paucity of receptors on the axonal membrane for the BZDs to work on [63]. One study showed that, during SE, endocytosis/internalization of GABA type A postsynaptic receptors is accompanied by an increase in the number of excitatory *N*-methyl-D-aspartate receptors (NMDARs) per somatic synapse on dentate granule cells. It is postulated that the decrease in GABA receptors with simultaneous upregulation of NMDARs may in part be the reason that BZDs fail to work in prolonged SE leading to RSE [64].

Early administration of benzodiazepines has been associated with better outcomes when studied in the prehospital setting in the randomized, controlled Prehospital Treatment of Status Epilepticus (PHTSE) trial. The trial showed that both diazepam and lorazepam were an effective prehospital treatment for seizures, as compared with placebo with early termination in 59.1% of patients receiving 4 mg IV lorazepam, 42.6% of those receiving 10 mg IV diazepam, and 21.1% of those receiving IV placebo [65]. Establishing intravenous access in patients who are having seizures in the prehospital environment can be challenging and time-consuming. The RAMPART trial compared IM midazolam (10 mg) to IV lorazepam (4 mg) in the prehospital status epilepticus setting. This study showed a lower rate of endotracheal intubation and recurrent seizures with IM midazolam administered through an autoinjector compared to IV lorazepam, thus proving that the IM route is safe and effective and can be considered as an alternative for prehospital treatment of convulsive seizures [66]. However, inadequate BZD dose by first responders continues to be a problem possibly leading to increased conversion to RSE, especially NCSE [67].

In a patient with SE, a second-line agent (IV AED) should be started at the onset as well, by giving a loading dose. The agents of choice are phenytoin (PHT)/fosphenytoin, valproic acid (VPA), levetiracetam (LEV), and lacosamide (LCM) [68]. There is no clear evidence that one drug is superior to another [69]. LEV has been studied extensively and has proven to be useful in SE [70–73]. It has a good safety profile which has made it the first-line AED for many providers. However, one retrospective study reported that VPA was better than LEV and PHT in controlling SE [74]. There are also good data for the use of VPA in SE, and it has been studied in six randomized controlled trials (RCTs) showing good efficacy [75–80]. The relative efficacy of VPA, LEV, and the other second-line treatments for SE (phenytoin and phenobarbital) has been assessed in a systematic review with meta-analysis [81]. Efficacy of LEV (68.5%) and VPA (75.7%) were found to be comparable with that of phenobarbital (73.6%) and higher than that of PHT (50.2), suggesting that LEV and VPA may represent valid alternatives to phenobarbital and PHT as second-line treatments of SE. One direct and indirect comparison of meta-analysis of LEV versus PHT or VPA for convulsive SE showed no difference between any two AEDs [82]. LCM is a relatively newer agent, and several studies have found it to be effective, and one study showed it to be even better than VPA [83, 84].

Overall, there is no single best second-line IV AED, and a drug may be chosen based on the treating provider's clinical experience and if the patient is already on one of these medications for chronic epilepsy. LEV and PHT (or fosphenytoin) tend to be the most common second-line IV AED.

### 5.2. Fourth-Generation AEDs

Although intravenous formulations are preferred for their fast onset of action, oral medications have been tried for RSE. Amongst the oral formulations, the ones which can be used in patients with SE are clobazam (CLB), perampanel (PER), topiramate (TPM), oxcarbazepine (OXC), and eslicarbazepine (ESL). CLB has been studied in patients with RSE as add-on therapy and found to be effective in terminating RSE [85, 86]. PER was marginally effective in a study by Rohracher et al. [87]. Similarly, TPM has been used as an add-on for RSE [88] but was not effective as monotherapy [89]. Kellinghaus et al. reported that OXC was effective in RSE after the failure of first- and second-line agents but required frequent electrolyte monitoring due to hyponatremia [90]. Brivaracetam was found to be effective in terminating SE in one study in Germany [91].

### 5.3. Other Medications Used as AEDs

RSE requires the administration of intravenous anesthetic drugs (IVADs) in the form of propofol (PRO), midazolam (MDL), or pentobarbital (PTB). Treatment of RSE has not been studied prospectively, and guidelines give a variety of options. In a systematic review comparing these three agents, PTB was efficacious and was associated with a lower frequency of short-term treatment failure, breakthrough seizures, and a change to a different IVAD. However, it was also associated with a higher frequency of hypotension which reflects the strong negative cardiovascular inotropic effect [92–94].

The administration of IVADs is typically associated with continuous EEG monitoring. Titration is done to achieve either seizure cessation or background suppression with the goal of EEG burst suppression patterns. In the same systematic review as above, compared with seizure suppression (30% of patients), titration of treatment to EEG background suppression (45% of patients) was associated with a significantly lower frequency of breakthrough seizures (4 versus 53%) and a higher frequency of hypotension (76 versus 29%). When aiming for burst suppression, the characteristic of the bursts is a better predictor of success in termination of RSE [95, 96]. While one theory suggests that burst suppression allows for the brain to rest, recover, and suppress the epileptiform activity, the disadvantage might be a worse outcome overall due to the need to use anesthetics and resulting prolonged intubation and hospitalization [11].

Ketamine's success in the treatment of RSE has been established in several studies and ranges from 32 to 73% [33, 97–99]. The ketamine's unique mechanism of action is through *N*-methyl-D-aspartate (NMDA) blockade, which in animal models has been demonstrated to be effective in prolonged SE when glutamatergic excitation is enhanced [100]. Additionally, ketamine tends to be more hemodynamically stable with protective properties in concomitant traumatic brain injury [101, 102].

Allopregnanolone is an endogenous neurosteroid with potent GABA modulation which demonstrated anticonvulsant properties in animal models [103]. In humans, brexanolone (SAGE 547) is an injectable allopregnanolone formulation used in the treatment of refractory status epilepticus in human patients [104]. Larger trials have demonstrated tolerability of brexanolone without demonstrable efficacy [105].

### 5.4. Immunotherapy

Treatment of RSE with immune etiology should follow the usual route with adequate dosing of abortive therapy with benzodiazepines followed by appropriately AEDs and IVADs. However, if an autoimmune cause is suspected especially if supported by the presence of autoantibody, prompt treatment with immunomodulating treatment is warranted. Early use of immunomodulating therapy may be associated with favorable outcomes. Considering progressive atrophy of the brain structures involved in status epilepticus on follow-up MRI, early aggressive therapy seems more appropriate. Though there may be increasing willingness to try immunotherapy early, there is no consensus or good quality data to suggest that one particular medication or therapy is better than others. Various immunotherapies are suggested and summarized in Table 5 (adapted from Zaccara et al.) [36, 37, 106].

One can start with IVIg or high-dose pulse corticosteroid therapy when an autoimmune etiology is suspected in case of RSE [107–110]. Initial laboratory evaluation to look for serum and CSF autoantibodies should be completed before initiation of immunomodulating therapies. If first-line treatment fails, one can consider either additional doses of the first-line treatment or PLEX [106, 111]. However, if IVIg is used initially, deploying PLEX is likely to negate its effects as it is likely to wash out immunoglobulins given prior. There is experience with various second-line therapies for the treatment of autoimmune encephalitis with neurological manifestations including seizures. However, in individual case series, there are very few patients with status epilepticus. Hence, the usefulness of the information for acute treatment of status epilepticus is limited. There are no systemic studies of using long-term immunotherapy for individuals who have autoimmune encephalitis with epilepsy. There is ample variability across the different case series with varying approaches and agents. At this point, the timing of the use of second-line immunomodulating agents in the management of SRSE is unclear. Their role in long-term management is more established, although the selection of an agent is on a case-by-case basis [44, 112–114]. Second-line agents are likely to take a longer time to produce the desired immunological response and are suited for chronic management of the underlying immunological dysfunction. This approach has limited application in the treatment of the acute setting of SRSE. On a different note, Rasmussen encephalitis, a childhood syndrome of refractory partial status epilepticus with presumed autoimmune etiology, is often treated with immunotherapy (chronic steroids, IVIg, or other immunosuppressive agents) or with hemispherectomy [115, 116].

### 5.5. Alternative Treatment

There is likely a significant publication bias for the following infrequently used treatment modalities.

There are 6 case reports described in the literature of vagus nerve stimulator (VNS) which is being used successfully in the treatment of SE. These included two children and six adults. It was effective in both generalized and focal SE. There was wide variability when the VNS was used ranging from 11 days to 14 months. However, all cases used a rapid increase in the VNS current and duty cycle. Efficacy varied between more than 50% improvement and seizure freedom. It was tolerated very well [117–122].

There have been 14 case reports and one recent case series of 8 patients with ECT use in the treatment of SE [123, 124]. Conventional thinking suggests that seizure induction during ECT is necessary for the cessation of SE; however, various cases have demonstrated that subconvulsive stimuli might be effective or even seizure induction might fail. From the published reports, there seems to be a success rate of approximately 70% for initial SE cessation. In the case series by Ahmed et al., ECT was initiated 7 to 39 days after onset of SE, and the patients underwent between 3 and 7 sessions guided by clinical judgment [123].

There are three documented case reports of the use of deep brain stimulation (DBS) in the treatment of SE. One patient with Rasmussen encephalitis of the left hemisphere origin and resulting epilepsia partialis continua intractable to immunotherapy was successfully treated with left caudal zona incerta (CZi) DBS [125]. Two other cases had bilateral DBS with leads placed in the centromedian nucleus (CMN): both of whom had the cessation of SE, but one patient who had cardiac arrest had poor clinical outcome [126, 127].

The ketogenic diet has been used in the treatment of refractory epilepsy in children for decades. While there is more experience of using diet therapy for treatment of SE in children [128, 129], it has recently been used in adults [130–138]. Ketogenic and modified Atkins diets lead to ketosis which controls seizures for unclear reasons. Ketosis likely also has some anti-inflammatory properties. Fat to carbohydrate and protein ratio of 4 : 1 or 3 : 1 is used. Across published case reports and series of 26 adult patients, diet therapy was started between days 2 and 60 of SE. It can take up to 16 days for ketosis to achieve, and the response can take up to 31 days since the onset of therapy but is less likely to occur after 14 days. Overall, the outcome is good with the resolution of SE in most cases that achieve ketosis although functional outcome can be variable [139]. In the largest recent prospective study of 15 patients, acidosis and hyperlipidemia seem to be the most common side effects leading to discontinuation of therapy in 3 patients. In the same study, few patients had switched to the modified Atkins diet by the time of long-term follow-up of 6 months [133].

Hypothermia not only produces electrocerebral silence [140] but may also be useful in treating RSE [141]. Experimental evidence further supports hypothermia's significant anticonvulsant properties [142–144]. Hypothermic rats demonstrated reduced epileptic brain damage related to SE when compared to normothermic and hyperthermic groups. Cooling, particularly in conjunction with diazepam, diminished the amplitude and frequency of epileptic discharges that translated into an anticonvulsant effect in rats tested [144]. The anticonvulsant mechanism by which hypothermia works is not fully understood. Hypothermia reduces excitatory transmissions, decreases the global cerebral metabolic rate of glucose and oxygen, reduces ATP breakdown, and stimulates glycolysis by intracellular alkalization enhancing energy production [143, 145]. Despite the ample data supporting hypothermia as both an effective neuroprotective agent and a powerful anticonvulsant, it remains unclear whether its use will translate into improved outcomes for patients with RSE [146].

### 5.6. Surgery

Surgical interventions for the treatment of RSE include acute resective surgery and disconnection procedures such as multiple subpial transection or corpus callosotomy [147]. Outcome data in acute status surgery are based on case reports and small series and present some publication bias. However, when pooled in a literature review, 56% of both adult and pediatric patients who underwent surgery for treatment of RSE were seizure free, and 31.4% had improvement in seizure frequency [148]. In pediatric patients, malformation of cortical development is the most common etiology (58.3%) of RSE, for which surgery has been commonly employed; in adults, the etiology varied and had variable outcomes [148]. Success was observed when surgery was done early (within one week) or later (greater than one month) [149, 150]. Unilobar lesion on MRI and congruency with EEG appear to correlate with a better outcome based on case reports and larger series, and patients with an inflammatory etiology do not do as well with acute status surgery which highlights the importance of a preoperative workup before the decision to consider a palliative surgical option [148].

**Figure 7:**
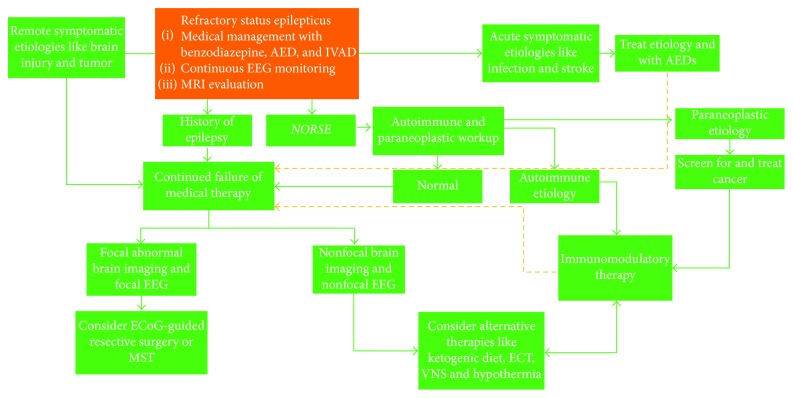
Flowchart depicting various options available in management of RSE and their suggested order. AED = antiepileptic drug, ECoG = electrocorticography, ECT = electroconvulsive therapy, IVAD = intravenous anesthetic drug, MST = multiple subpial transections, NORSE = new-onset refractory status epilepticus, and VNS = vagus nerve stimulator.

Various steps and options proposed for management of RSE are depicted in Figure 7.

## 6. Prognostic Factors and Outcomes

Many studies have looked at prognostic factors of SE overall and did not specifically obtain data for RSE or SRSE, so these studies will have an admixture of relatively better prognosis for SE and worse prognosis for SRSE.

The underlying etiology of RSE seems to be the most frequent and important prognostic factor. Stroke-induced RSE has a poor prognosis and high mortality [151]. In another study, postanoxic encephalopathy and brain tumors were independently associated with the increased rate of death [20]. A previous history of epilepsy was associated with poor outcome in one study but not in another [20, 152].

Lower levels of consciousness (coma or stupor) at the onset of SE are more likely to result in mortality. Also, GCSE and NCSE were independently associated with death [20]. Duration of RSE and duration of coma greater than ten days also have an unfavorable outcome [11, 152]. On the other hand, there have been reports of survival even after severely prolonged SE [153]. EEG findings of periodic epileptiform discharges are more frequently associated with RSE [19]. On the contrary, the absence of burst suppression and isoelectric EEG is associated with good outcome possibly due to the reduced burden of anesthetic medications and decreased duration of coma and hospitalization [11]. Low levels of albumin at onset are independently associated with RSE and death as per one study [154]. Reduction or withdrawal of AEDs is likely not going to result in RSE [3, 155]. Various prognostic factors from selected studies are noted in Table 6.

Short-term mortality in adults ranges from 9% in SE to 38% in RSE [20, 156, 157]. Status epilepticus severity score (STESS) was developed to assess short-term mortality and comprises variables of consciousness impairment, worst seizure type, age, and history of seizures. Stupor or coma, NCSE, and age greater than 64 years were considered poor outcome factors, while a history of previous seizures was considered a good outcome factor. A score of two or less is supposed to have a good short-term outcome [158], but a score greater than two has low specificity for poor outcome. Addition of modified Rankin scale to STESS and named mSTESS has been proposed. Based on one study, mSTESS has better positive predictive value (PPV) than STESS at scores greater than 3. An mSTESS has a PPV of 81.8% for short-term mortality as compared to 59.6% for the STESS [159].

In an extensive review of therapies in 596 convulsive RSE and SRSE cases, assessment of long-term outcomes showed that approximately 35% of cases reached baseline neurological status, 35% died, and 30% had variable neurological deficits. The duration at which outcome was assessed varied from months to years [107]. Since that review, multiple studies of RSE (convulsive and nonconvulsive) with cases numbering less than 100 have been published with a similar long-term outcome—recovery to baseline in 36%, neurological deficit in 23%, and death in 41% [23].

## 7. Conclusion

SE and its more severe forms RSE and SRSE continue to be a significant management challenge. NORSE tends to have autoimmune and paraneoplastic etiologies commonly, but clarity in testing and management protocols is lacking. Clinicians and patients would also benefit from a comprehensive meta-analysis of prognostic factors as currently different studies show variable results. Also, studies dedicated to management and outcome in special populations including elderly, pregnant females, and those with neurodegenerative diseases are lacking. There is also a need for large multicenter trials for early prediction models for SE and how different predictive factors should be weighted. Future studies should aim to tackle these issues.

## Figures and Tables

**Table 1:** Etiologies of status epilepticus.

*Broadly defined etiologies of status epilepticus*
Known
(i) Acute
(ii) Remote
(iii) Progressive
(iv) In defined electroclinical syndromes
Unknown
*Specific etiologies of status epilepticus*
Cerebrovascular diseases
CNS infections
Neurodegenerative diseases
Intracranial tumors
Cortical dysplasias
Head trauma
Alcohol related
Intoxication
Withdrawal of or low levels of AEDs
Cerebral hypoxia or anoxia
Metabolic disturbances
Autoimmune disorders
Mitochondrial diseases

**Table 2:** Etiology of RSE in selected studies.

Study	*N*	Known (%)			Unknown (%)
		Acute	Remote	Progressive	
Delaj et al. (RSE versus NRSE)^ [21]	RSE = 301	58.5	12.6^#^	20.9	8.6
Delaj et al. (RSE versus SRSE)^ [21]	RSE = 268 SRSE = 33	51.6	15.2	18.2	9
Holtkamp et al. [3]	36	50^∗^	22.2	16.7	0
Giovannini et al. [10]	26	77^∗^	12	4	0
Kantanen et al. [16]	75	41	51	5	3

^#^NRSE was significantly more likely to have a remote etiology as compared to RSE; ^∗^RSE was significantly more likely to have an acute etiology as compared to NRSE; ^Delaj et al. differentiated RSE and SRSE cases in their cohort (RSE = refractory status epilepticus and NRSE = nonrefractory status epilepticus).

**Table 3:** Distribution of specific etiologies of RSE in selected studies.

Study	*N*	Unknown	Cerebrovascular disease	CNS infections				Intracranial tumor	Head trauma	Substance related			Hypoxic/anoxic brain injury	Metabolic disturbances	Autoimmune/immunological conditions	Sepsis/systemic infections
				Encephalitis	Meningitis	Others	Total			AEDs	Others	Total				
Ferlisi et al. (audit) [24]	478	20	13	13	3	7	23	5	4	8	5	13	11	5	6	0
Holtkamp et al. [3]	36	0	30	22^∗^	0	0	22	8	0	0^#^	11	11	11	6	8	0
Vooturi et al. [12]	45	11	18	31^∗^	9	4	44^∗^	0	0	9	7	16	0	2	0	0
Giovannini et al. [10]	26	0	12	0	0	0	0	8	0	0	0	0	50^∗^	8	0	12
Hocker et al.^1^ [11]	63	4.8	11	—	—	—	11	9	0	16	3	19	0	11	8	9
Gaspard et al. [5]	130	52	0	—	—	—	8	0	0	0	0	0	0	0	37	0
Kantanen et al.^1,3^ [16]	75	4	12	—	—	—	4	3	15	0	17	17	0	3	0	0
Sutter et al.^3^ [20]	111	9	13	—	—	—	7	14	6	0	3	13	23	4	0	0

^1^Hypoxic/anoxic brain injury excluded; ^2^NORSE cases only; ^3^preexisting epilepsy in 32% of cases in Kantanen et al. and 10% of cases in Sutter et al.; ^∗^statistically significant etiology of RSE as compared to NRSE; ^#^statistically less likely etiology of RSE as compared to SE; NORSE = new-onset refractory status epilepticus, RSE = refractory status epilepticus, and NRSE = nonrefractory status epilepticus.

**Table 4:** Diagnostic investigations in RSE. Adapted from the NORSE table of investigations on http://www.norseinstitute.org/definitions/. This is the basic workup suggested to be done in most patients with NORSE and is by no means an absolute list. For further workup and a complete list of tests, please refer to the NORSE Diagnostic Checklist which can be found on http://www.norseinstitute.org/definitions/ [25].

Basic workup for causes of refractory status epilepticus	
Screen	Disease/agent tested

Infectious	Recommended in most or all patients
	(i) Serologic: bacterial and fungal cultures, RPR-VDRL, and HIV-1/2 immunoassay with confirmatory viral load if appropriate
	(ii) CSF: cell counts, protein, glucose, bacterial and fungal stains and cultures, VDRL, PCR for HSV1, HSV2, VZV, EBV, HIV, and *Mycobacterium tuberculosis*
	Recommended in immunocompromised patients in addition to above
	(i) Serologic: IgG *Cryptococcus* species, IgM and IgG *Histoplasma capsulatum*, and IgG *Toxoplasma gondii*
	(ii) Sputum: *Mycobacterium tuberculosis* GeneXpert
	(iii) Serum and CSF: *Toxoplasma* IgG
	(iv) CSF: eosinophils, silver stain for CNS fungi, PCR for JC virus, CMV, HHV6, EEE, *Enterovirus*, influenza A/B, WNV, *Parvovirus*, *Listeria* Ab, and measles (rubeola)
	(v) Stool: adenovirus PCR and *Enterovirus* PCR

Vascular	(i) CTA or MRA and MR venography

Autoimmune/paraneoplastic	Recommended
	(i) Serum and CSF paraneoplastic and autoimmune epilepsy antibody panel
	To include antibodies to VGKC with LGI-1 and CASPR2, Ma2/Ta, DPPX, GAD65, NMDA, AMPA, GABA-B, GABA-A, glycine receptor, amphiphysin, CV-2/CRMP-5, neurexin-3 alpha, adenylate kinase, anti-neuronal nuclear antibody types 1 (Hu), 2 (Ri), and 3, Purkinje cell cytoplasmic antibody types 1 (Yo), Tr, and 2, and glial nuclear antibody type 1
	(ii) Serologic: also send for ANA, ANCA, anti-thyroid antibodies, anti-dsDNA, ESR, CRP, ENA, SPEP, and IFE. Antibodies for Jo-1, Ro, La, Scl-70, RF, and ACE; anti-tTG and anti-endomysium antibodies and cold and warm agglutinins
	Optional: consider storing extra frozen CSF and serum for possible further autoimmune testing in a research lab

Neoplastic	Recommended: CT chest/abdomen/pelvis, scrotal ultrasound, mammogram, CSF cytology, flow cytometry, and pelvic MRI
	Optional: bone marrow biopsy, whole-body PET-CT, and cancer serum markers

Metabolic	Recommended: LDH and ammonia
	Considered: vitamin B1 level, B12 level, folate, lactate, pyruvate, CPK, and troponin; tests for mitochondrial disorder (lactate and pyruvate); serum triglycerides

Toxicological	Recommended: benzodiazepines, amphetamines, cocaine, fentanyl, alcohol, ecstasy, heavy metals, synthetic cannabinoids, and bath salts
	Considered: extended opiate and overdose panel, LSD, heroin, PCP, and marijuana

Genetic	Considered: genetics consult, ceruloplasmin, and 24-hour urine copper

**Table 5:** Immunomodulating treatment.

*First*-*line immunotherapies*
PLEX
Dosage: various numbers of plasma exchanges reported, typically 5 sessions of plasma exchange
Advantages: no long-term immunosuppressive effect
Disadvantages: requires large lumen intravascular indwelling catheter placement increasing chances for line sepsis and procedure-related complication and hemodynamic effect of PLEX can be detrimental in a patient with hypotension due to IVAD use
Corticosteroids
Dosage: various dose regimens reported in literature. Most commonly used regimen is IV methylprednisolone 1 g daily for 5 days followed by weekly single administration of 1 g for 4–6 weeks or conversion to oral prednisone 80 mg/day with a slow taper
Advantages: easily available, relatively inexpensive, and familiarity with the drug
Disadvantages: increases blood pressure, may increase vulnerability for infection, and may worsen hyperglycemia in patients with diabetes mellitus
IVIg
Dosage: 0.4 g/kg daily for 3–5 days and can be repeated weekly/monthly for 1–3 months
Advantages: no immunosuppressive effect
Disadvantages: allergy; increased volume load may worsen congestive heart failure; increased risk of thrombotic events such as deep vein thrombosis and pulmonary embolism and risk of renal function impairment especially in the presence of renal artery stenosis may cause aseptic meningitis presenting as headache and allergy
*Second*-*line immunotherapies*
Cyclophosphamide
Dosage: 750 mg/m^2^
Advantages: well-known drug with a long track record which can be used by administrating monthly
Disadvantages: may not be immediately effective (suitable for maintenance therapy), may increase the risk of infections, has teratogenic potential, may increase the risk of future malignancy, and side effects include hemorrhagic cystitis, severe cardiotoxicity, alopecia, and nausea/vomiting
Rituximab
Dosage: most commonly used dose is 375 mg/m^2^ every week for 4 weeks
Advantages: usually well tolerated
Disadvantages: may not be immediately effective and may cause cytopenia, infusion reaction, potential for severe allergic reaction, renal failure, pregnancy, and hepatitis
Mycophenolate
Dosage: 250 mg–2 g per day (no standard dosing for autoimmune encephalitis)
Advantages: oral preparation for long-term use, usually well tolerated
Disadvantages: may not be immediately effective (suitable for maintenance therapy), needs oral administration, may be difficult in the ICU setting, may cause significant gastrointestinal side effects and hyperglycemia, and highly protein bound so may interact with AEDs that are protein bound
Azathioprine
Dosage: 1–3 mg/kg per day
Advantage: oral preparation for long-term use, usually well tolerated, and can be used as a steroid-sparing agent
Disadvantage: side effects such as elevated hepatic transaminases, leukopenia, pancreatitis, and immunosuppression

**Table 6:** Long-term outcome factors for RSE in selected studies.

Study	Older age	STESS >2	History of epilepsy or status epilepticus	Longer duration	Sepsis/systemic infection	Baseline functioning	EEG findings (no BS or isoelectric EEG)	Seizure or status epilepticus type	Etiology category	Cardiac arrythmia	Long duration of mechanical ventilation	Need for CPR
Kantanen et al. [23]	↓	NE	NE (epilepsy)	NE	NA	NE	NA	NE	NE	NA	NA	NA
Madzar et al. [152]	↓	↓	↓(epilepsy)	↓^1^	↓	NE	NA	NE	NE	NA	NE	NA
Hocker et al.^2^ [11]	NE	NA	NE	↓^3^	↓^4^	NA	↑	NE	NA	↓	↓	NA
Sutter et al. [20]	NE	NA	NE	↓	NE	NA	NA	↓^5^	↓^6^	NA	NE	↓

↓, worse outcome; ↑, better outcome; NE, no effect; NA, not assessed or not available; CPR, cardiopulmonary resuscitation; BS, burst suppression; ^1^duration of RSE >10 days; ^2^anoxic brain injury etiology excluded; ^3^duration of coma >10 days; ^4^effect seen with pneumonia; ^5^effect seen with GCSE and NCSE; ^6^effect seen only with hypoxic/anoxic brain injury and intracranial tumor.
